# Supplementary material for: FoxP3 forms a head-to-head dimer in vivo and stabilizes its multimerization on adjacent microsatellites
Source: Cell Rep. Author manuscript; Available in PMC 2026 Jan 21. (PMC12820563; doi:10.1016/j.celrep.2025.116633)
Supplement: 1 [file NIHMS2132744-supplement-1.pdf]

Cell Reports, Volume *44*

Supplemental information

FoxP3 forms a head-to-head dimer *in vivo*

and stabilizes its multimerization

on adjacent microsatellites

Fangwei Leng, Ryan Clark, Wenxiang Zhang, Thibault Viennet, Cuidie Wang, Haribabu Arthanari, Xi Wang, and Sun Hur

SUPPLEMENTARY FIGURES

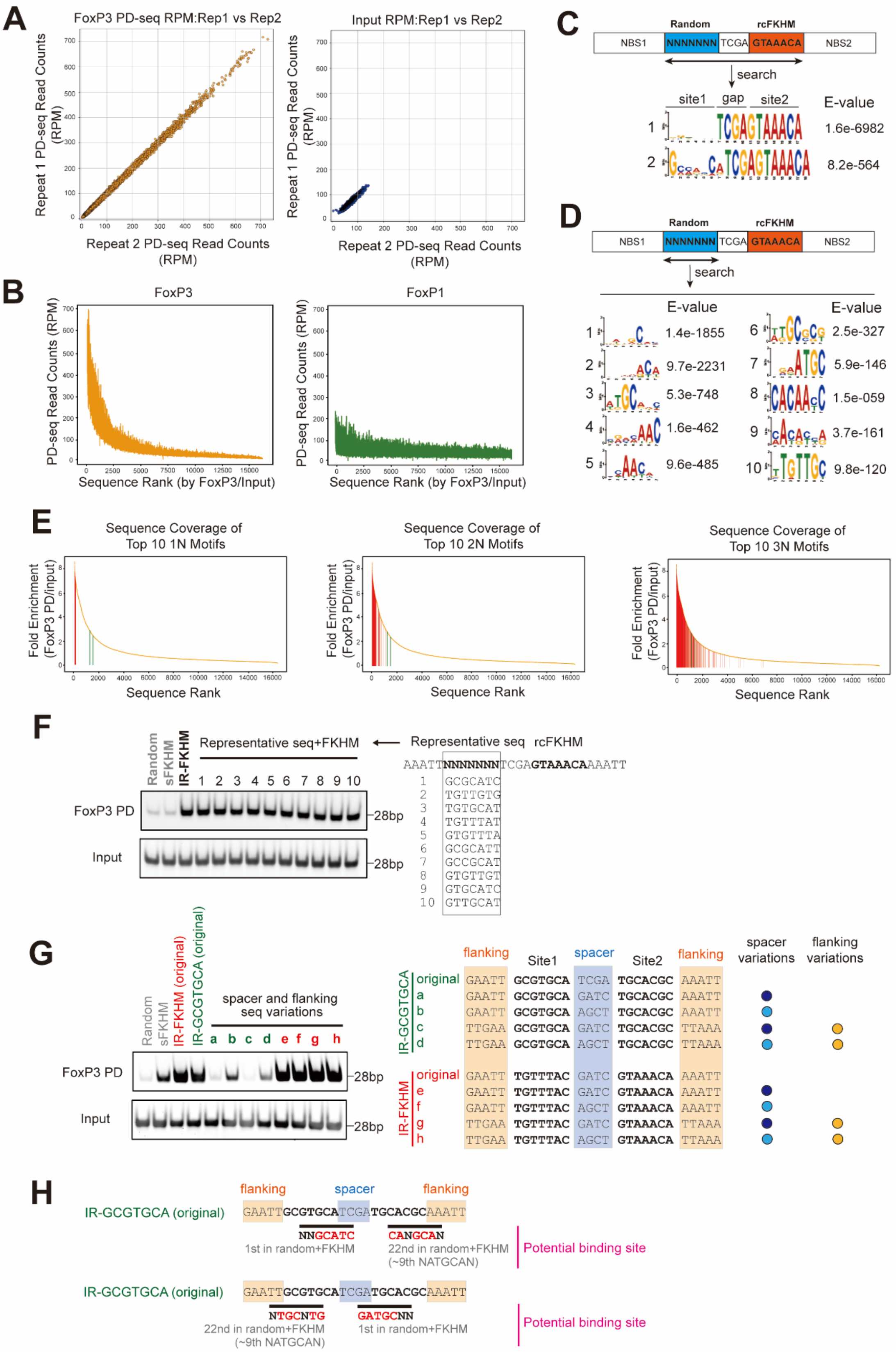

Figure S1. PD-seq analysis of FoxP3 and FoxP1. Related to Figure 1.

A. Scatter plots showing reproducibility between two biological replicates of FoxP3 PD-seq (left) and input control (right). Read counts (in RPM) for replicate 1 and replicate 2 are plotted on the x- and y-axes, respectively, demonstrating high concordance for FoxP3 PD-seq.
B. PD-seq read count distributions for FoxP3 (left) and FoxP1 (right), with sequences ranked in descending order of FoxP3/Input enrichment (same rank order as in Figure 1B). FoxP3 RPM values show a steep decline, mirroring the enrichment distribution and confirming that highly enriched sequences are also abundant in the pull-downs ample. In contrast, FoxP1 RPM values are more uniform across the same sequence ranks, suggesting low selectivity for site1.
C. De novo motif discovery using MEME to search sequences covering the 14 nt region encompassing site 1, the gap and site 2 (rcFKHM) failed to capture the variable sequences in site 1 due to overwhelming signal from the fixed sequence in the gap and site 2.
D. De novo motif discovery using MEME on sequences containing only site 1.
E. Rank-ordered enrichment plots (in descending order of FoxP3/Input enrichment) where sequences matching the top 10 motifs with degenerate bases at 1, 2, and 3 positions (1N, 2N, and 3N motifs) were indicated with red lines. Allowing 2N degeneracy achieves broad coverage of top-enriched sequences (top 10% of enrichment values) while maintaining resolution, which justifies its use in our motif analysis. Green lines mark sequences matching the putative GCGYGCH motif identified from the random–random library (Table S2), which was not enriched in the random–rcFKHM library.
F. FoxP3 PD was performed using MBP-tagged FoxP3$^{\Delta N}$ (0.4 μM) to compare its binding affinity to DNA (0.1 μM) containing representative sequences of the top 10 2N-motifs, each paired with the reverse complement of FKHM (rcFKHM, GTAAACA). Numbers next to individual motifs indicate rank orders based on fold enrichment in Figure 1E.
G. Spacer and flanking sequence requirements for IR-GCGTGCA vs. IR-FKHM binding. DNA constructs containing IR-GCGTGCA (green) or IR-FKHM (red) were tested in FoxP3 PD assays, performed as in (F). "Original" sequences correspond to motifs with spacer and flanking regions derived from the random-random library. Mutations in the spacer or flanking sequences abolished FoxP3 binding to IR-GCGTGCA, whereas IR-FKHM binding was largely unaffected.
H. Schematic showing that IR-GCGTGCA within the random–random library harbors two inverted pairs of high-ranking motifs from Figure 1 at ±2 shifted positions. These overlapping motifs likely account for the apparent enrichment of IR-GCGYGCH.

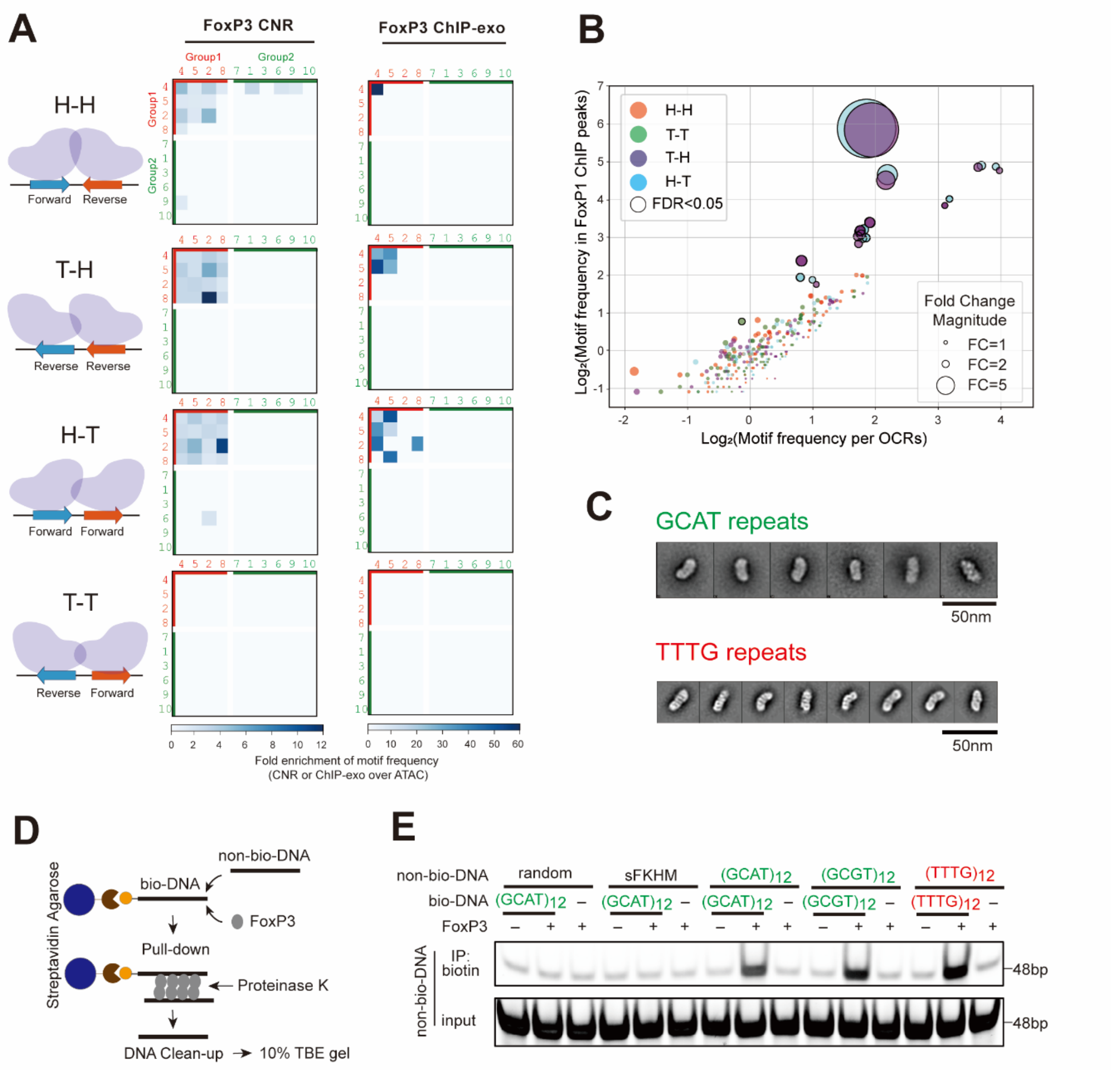

Figure S2. FoxP3 recognizes both H-H motifs and TnG repeats in Tregs. Related to Figure 3.

A**.** Heatmaps showing fold change of the motif frequencies in FoxP3-bound sites over OCRs, grouped based on the motif pair orientation. The top 10 motifs are further categorized into group 1 (G1, red) and group 2 (G2, green) sequences. FoxP3 CNR-seq data are from Tregs (left) and ChIP-exo from mESCs (right). Only the motif pairs that are significantly enriched in FoxP3-bound sites ($p<0.05$, exact binomial test) are shaded.

B. Log2-transformed motif frequencies in FoxP1 ChIP-seq peaks (35,767 peaks) vs. OCRs (based on ATAC-seq, 502,437 peaks). Each point represents one of the 400 motif pairs (10 motifs x 10 motifs x 4 orientation combinations) with a 4-nt gap, colored by the motif orientation relative to the reference genome (positive strand): H-H, T-H, H-T, and T-T. Motif frequency was defined as the number of motif occurrence within all ChIP-peaks (or ATAC-

peaks) divided by the total length (in million bp) of those peaks. Dot size reflects fold change of motif frequency in ChIP-peaks over OCRs. Motif pairs significantly enriched in FoxP3-bound regions ($p<0.05$, exact binomial test) are outlined in black and are further analyzed in Figure 3B.

C. Representative 2D class averages from negative-stain electron microscopy of FoxP3$^{\Delta N}$ in complex with $(GCAT)_{15}$ (top) and $(TTTG)_{18}$ (bottom). FoxP3 multimers form on both TTTG and CGAT repeats with similar morphologies. Consistent with DNA length, FoxP3 multimers on both sequences measure ~25 nm.

D. Schematic of DNA bridging assay. Biotinylated DNA (bio-DNA, 82 bp), pre-conjugated to streptavidin agarose beads, and non-biotinylated DNA (non-bio-DNA, 48 bp) were mixed at a 1:1 ratio (0.1 μM each), incubated with FoxP3$^{\Delta N}$ (0.4 μM) and subjected to pull-down. Co-purified non-bio-DNA was then recovered by proteinase K digestion and analyzed on TBE gels (see Methods for details).

E. DNA bridging assay using indicated bio-DNA and non-bio-DNA sequences.

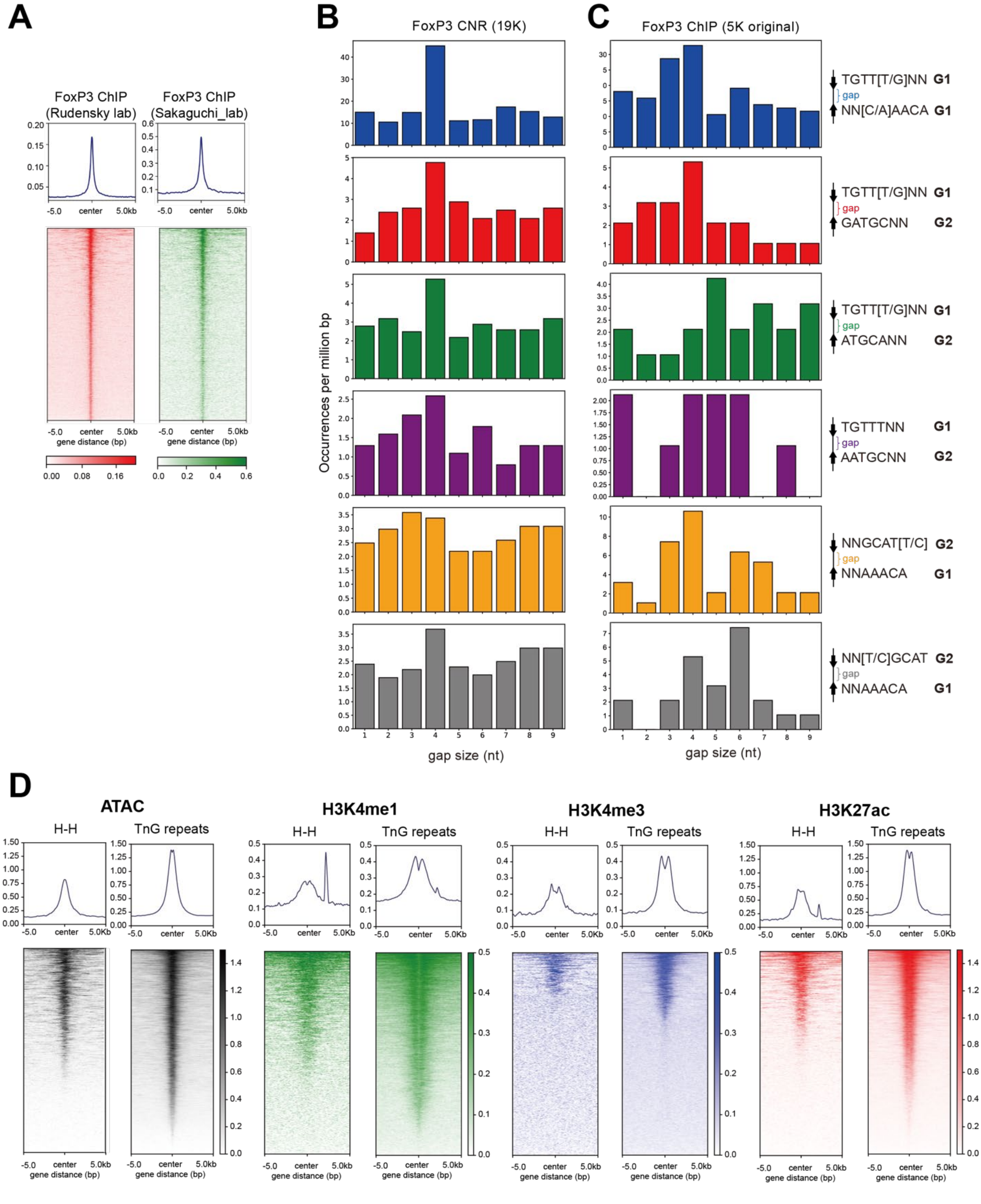

A
FoxP3 ChIP (Rudensky lab)
FoxP3 ChIP (Sakaguchi_lab)
gene distance (bp)
B
FoxP3 CNR (19K)
C
FoxP3 ChIP (5K original)
Occurrences per million bp
gap size (nt)
TGTT[T/G]NN G1
gap
NN[C/A]AACA G1
TGTT[T/G]NN G1
gap
GATGCNN G2
TGTT[T/G]NN G1
gap
ATGCANN G2
TGTTTNN G1
gap
AATGCNN G2
NNGCAT[T/C] G2
gap
NNAAACA G1
NN[T/C]GCAT G2
gap
NNAAACA G1
D
ATAC
H3K4me1
H3K4me3
H3K27ac
H-H
TnG repeats

Figure S3. Analysis of FoxP3-bound H-H motifs in Tregs. Related to Figure 4.

A. FoxP3 ChIP-seq signal across ±5 kb centered on H-H motif-containing loci in Tregs from Rudensky lab (PMID:23021222) and Sakaguchi lab (PMID: 29044238). Each row represents a genomic region, ranked using the same order in both heatmaps. Average signal profile is shown above each heatmap.
B. Gap size distributions for the indicated motif pairs across FoxP3 CnR-seq peaks (n=18,552).
C. Gap size distributions for the indicated motif pairs across the original FoxP3 ChIP-seq peaks (n=5,047) used in PMID: 35926508.
D. Signal intensities for ATAC-seq and histone ChIP-seq (H3K27ac, H3K4me1, and H3K4me3) across ±5 kb centered on FoxP3-bound TnG repeats (n=19,145) and H–H motifs (n=2,375). Each row represents a genomic region. Average signal profile is shown above each heatmap.

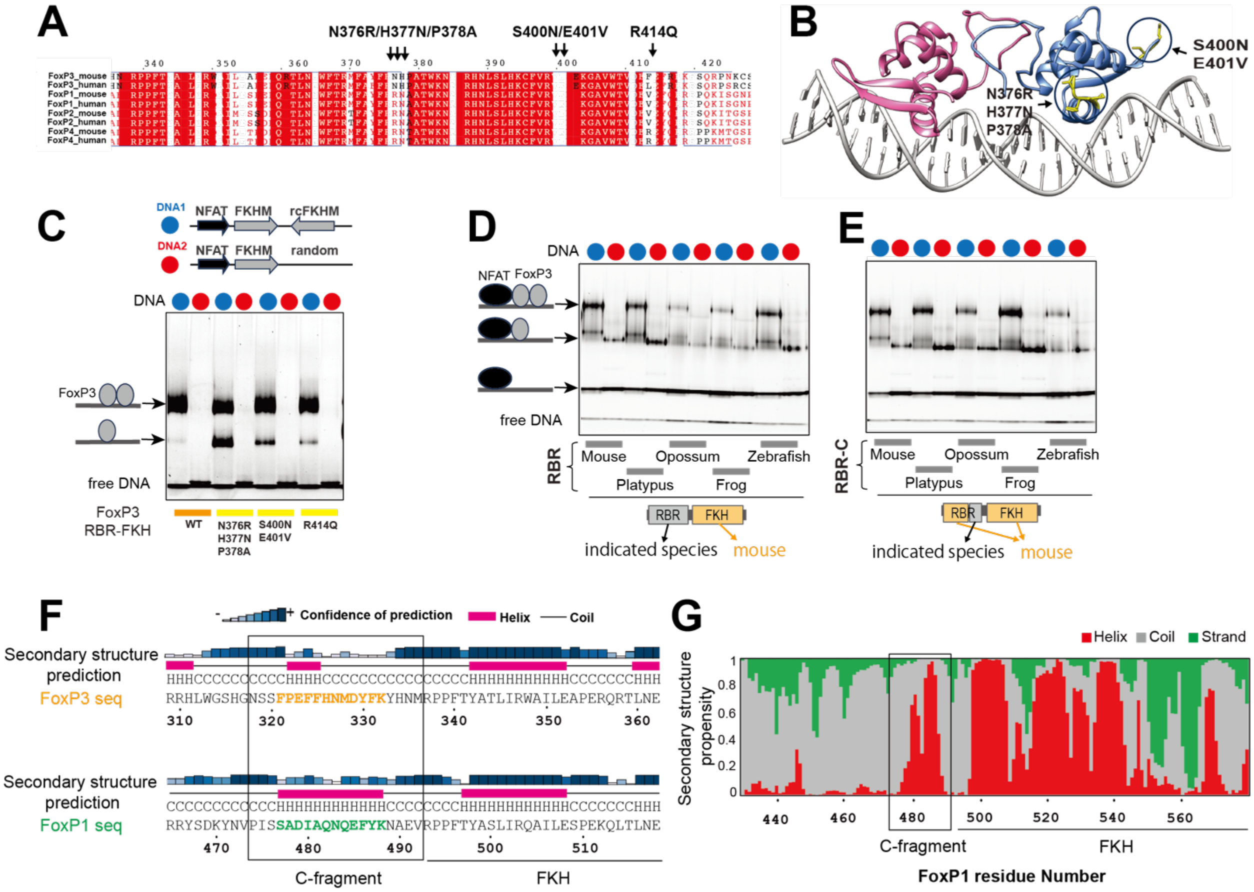

Figure S4. RBR-C is the key determinant for FoxP3 H-H dimerization. Related to Figure 6.

A. Sequence alignment of the FKH domain of mouse and human FoxP TFs, with moderately conserved residues indicated in red font and highly conserved residues highlighted in red. Arrows indicate divergent residues between FoxP3 and FoxP1/2/4.

B. Structure highlighting the divergent residues in the FKH domain indicated in (A).

C. Native gel shift assay of WT and mutant FoxP3$^{RBR\text{-}FKH}$ (0.8 µM, NusA-tagged) with IR-FKHM (blue) or sFKHM (red) DNA (0.2 µM). Mutations in the divergent residues in (A) had minimal impact on FoxP3's preference for IR-FKHM over sFKHM, although they commonly reduced FoxP3's dimerization on IR-FKHM to varying degrees.

D-E. Native gel shift assays using various chimeric RBR-FKH constructs, in which the RBR or RBR-C domain of mouse FoxP3 was replaced with that from different FoxP3 orthologs. FoxP3 (0.8 µM) was incubated with IR-FKHM (blue) or sFKHM (red) DNA (0.2 µM) in the presence of NFAT (0.4 µM). Although swapping the FoxP3 RBR loop with those from other species altered the overall DNA-binding affinity, it did not affect the preferred binding mode; all constructs bound IR-FKHM as a dimer and sFKHM as a monomer, mirroring the behavior of unswapped mouse FoxP3.

F. Secondary structure prediction (by PSIPRED) indicated an α-helix (purple) in the RBR-C region of FoxP1 (bottom, highlight in green), whereas FoxP3 RBR-C (top, highlight in yellow) was predicted to contain only a short, four–amino acid helical stretch.

G. NMR experiments indicated a strong α-helical propensity (red) for FoxP1 residues 477–488 within RBR-C fragment, consistent with the predicted secondary structure.
